# Influence of standard modifiable risk factors on ventricular tachycardia after myocardial infarction

**DOI:** 10.3389/fcvm.2023.1283382

**Published:** 2023-10-24

**Authors:** Tejas Deshmukh, Joshua G. Kovoor, Karen Byth, Clara K. Chow, Sarah Zaman, James J. H. Chong, Gemma A. Figtree, Aravinda Thiagalingam, Pramesh Kovoor

**Affiliations:** ^1^ Department of Cardiology, Westmead Hospital, Sydney, NSW, Australia; ^2^ Centre for Heart Research, Westmead Institute for Medical Research, University of Sydney, Westmead, Sydney, NSW, Australia; ^3^ University of Adelaide, The Queen Elizabeth Hospital, Adelaide, SA, Australia; ^4^ Research and Education Network, Western Sydney Local Health District, Westmead Hospital, Sydney, NSW, Australia; ^5^ Kolling Institute, Royal North Shore Hospital, Sydney, NSW, Australia; ^6^ Charles Perkins Centre, University of Sydney, Sydney, NSW, Australia

**Keywords:** myocardial infarction, ventricular tachycardia, electrophysiology study, standard modifiable risk factors, LVEF (left ventricular ejection fraction)

## Abstract

**Background:**

Inducible ventricular tachycardia (VT) at electrophysiology study (EPS) predicts sudden cardiac death because of ventricular tachyarrhythmia, the single greatest cause of death within 2 years after myocardial infarction (MI).

**Objectives:**

We aimed to assess the association between standard modifiable risk factors (SMuRFs) and inducible VT at EPS early after MI.

**Methods:**

Consecutive patients with left ventricle ejection fraction ≤40% on days 3–5 after ST elevation MI (STEMI) who underwent EPS were prospectively recruited. Positive EPS was defined as induced sustained monomorphic VT cycle length ≥200 ms for ≥10 s or shorter if hemodynamically compromised. The primary outcome was inducibility of VT at EPS, and the secondary outcome was all-cause mortality on follow-up.

**Results:**

In 410 eligible patients undergoing EPS soon (median of 9 days) after STEMI, 126 had inducible VT. Ex-smokers experienced an increased risk of inducible VT [multivariable logistic regression adjusted odds ratio (OR) 2.0, *p* = 0.033] compared with current or never-smokers, with comparable risk. The presence of any SMuRFs apart from being a current smoker conferred an increased risk of inducible VT (adjusted OR 1.9, *p* = 0.043). Neither the number of SMuRFs nor the presence of any SMuRFs was associated with mortality at a median follow-up of 5.4 years.

**Conclusions:**

In patients with recent STEMI and impaired left ventricular function, the presence of any SMuRFs, apart from being a current smoker, conferred an increased risk of inducible VT at EPS. These results highlight the need to modify SMuRFs in this high-risk subset of patients.

## Graphical Abstract

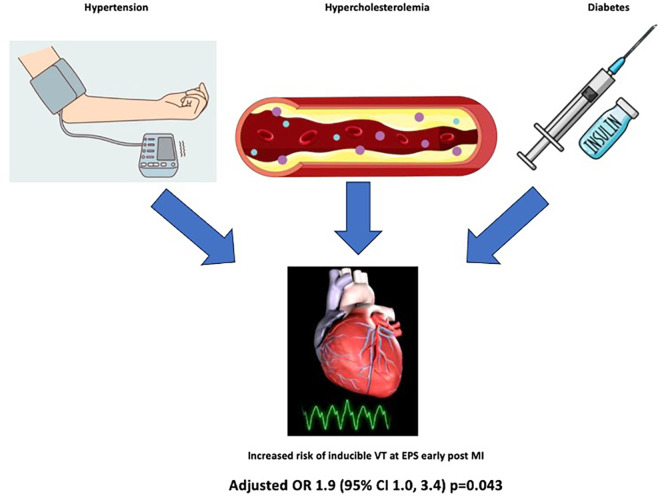

## Introduction

Patients without standard modifiable risk factors (SMuRFs) have reported higher all-cause mortality at 30 days after ST elevation myocardial infarction (STEMI) compared with those with at least one standard modifiable coronary artery risk factor, including hypertension, diabetes, hypercholesterolemia, and smoking (1–3). This was not driven by recurrent ischemic events, stroke, or bleeding, suggesting potential heightened susceptibility to fatal arrhythmia as the cause of the unexpected differences. Sudden cardiac death because of ventricular tachyarrhythmia is the single greatest cause of death within 2 years after myocardial infarction (MI), especially early in the first 30 days (4, 5).

Extensive work from our group has shown that in patients with left ventricular ejection fraction (LVEF) ≤40% early after reperfusion for STEMI, the presence of inducible ventricular tachycardia (VT) during electrophysiology (EP) studies (EPS) is a marker for the future occurrence of spontaneous ventricular tachyarrhythmias (6–8). No previous study has assessed the impact of the presence or absence of SMuRFs on the risk of inducible VT early after MI during EPS. We aimed to evaluate the incidence of inducible VT at EPS in individuals with LVEF ≤40% shortly after MI, comparing those with standard modifiable coronary artery risk factors to those without.

## Methods

### Study protocol

The protocol for the collection of data utilized for this study, comprising patient recruitment, follow-up and implantable cardioverter–defibrillator (ICD) implantation and programming, has previously been described in detail (6, 9–13). The Western Sydney Local Health District Human Research Ethics Committee has approved the study, and all patients gave their informed consent. Consecutive patients with LVEF ≤40% on days 3–5 after MI who underwent EPS were prospectively recruited.

A positive EPS was defined as sustained monomorphic VT with cycle length (CL) ≥200 ms (7, 12) for ≥10 s or shorter if hemodynamically compromised (11). Inducible ventricular fibrillation (VF) or ventricular flutter with CL <200 ms was considered non-inducible VT (9, 14). The full protocol for the programmed ventricular stimulation is provided in the Supplementary Material. Predischarge ICD implantation was recommended for patients with inducible VT at EPS (7, 8, 15).

### Statistical analysis

IBM SPSS Statistics version 28 was used to analyze the data. SMuRFs were defined as being a current smoker, hypercholesterolemia, diabetes, or hypertension as per the criteria set out in Figtree et al. (1) and also provided in the Supplementary Material.

The chi-squared test or exact permutation tests, when appropriate, were used to assess the univariable association between each categorical variable and VT at EPS status (present vs. absent). The Mann–Whitney test was used to evaluate the univariable association between VT at EPS and the continuous variables age and LVEF (%). LVEF (%), gender, previous coronary artery bypass grafting (CABG) status, and previous percutaneous transluminal coronary angioplasty (PTCA) status were the non-SMuRF variables demonstrating univariable association with VT at EPS at the *p *≤ 0.2 level. These variables were selected as candidates for inclusion along with either the number of SMuRFs (0–4) or the presence of any SMuRFs in multiple logistic regression models of VT at EPS. Adjusted odds ratios (ORs) and 95% confidence intervals (95% CIs) from the best-fitting logistic regression models were used to quantify the association of each variable with VT at EPS.

Multiple logistic regression models were fitted, incorporating LVEF (%), gender, previous CABG status, and previous PTCA status along with smoking history and either the number of other SMuRFs (0–3, excluding current smoking status) or the presence of any other SMuRFs as candidates for inclusion. Adjusted ORs with 95% CIs were reported.

Log-rank tests were used to assess the univariable association between each of the categorical variables and the right-censored outcome “years from EPS to death” [overall survival (OS)]. Cox proportional hazards (PH) models were used to assess the univariable association between OS and each continuous variable. The non-SMuRF variables demonstrating univariable association with OS at the *p *≤ 0.2 level were age, LVEF (%), family history of cardiovascular disease (CVD), previous ischemic heart disease (IHD), previous CABG, previous PTCA, previous cerebrovascular accident (CVA), and VT at EPS. These variables were selected as candidates for inclusion along with either the number of SMuRFs (0–4) or the presence of any SMuRFs in multiple Cox PH models. For the removal, backward stepwise variable selection with *p* > 0.1 was used to identify the best-fitting model containing the independent predictors of OS. Hazard ratios (HRs) with 95% CI were used to quantify the strength of the association. Two-tailed tests with a significance level of 5% were used throughout.

## Results

Tables 1A,B show the association between potential risk factors and VT at EPS in 410 participants.

**Table 1A:** Patient characteristics by VT at EPS status (categorical variables).

Variable	Values taken	No VT	VT at EPS	Percentage VT ve at EPS (%)	Chi-squared or Mann–Whitney* *p*-value
		(*n* = 284)	(*n* = 126)		
Sex	Female	53	15	22.1	0.087
	Male	230	111	32.6	
Family history of CVD	No	143	58	28.9	0.563
	Yes	128	59	31.6	
Previous IHD	No	214	84	28.2	0.201
	Yes	61	33	35.1	
Previous CABG	No	270	111	29.1	0.072
	Yes	4	6	60.0	
Previous PTCA	No	244	96	28.2	0.050
	Yes	31	22	41.5	
Previous CVA	No	265	114	30.1	1.000
	Yes	9	3	25.0	
LVEF ≤30%	>30%	196	68	25.8	0.003
	≤30%	87	58	40.0	
Elevated BP	No	152	52	25.5	0.050
	Yes	123	65	34.6	
High cholesterol	No	136	45	24.9	0.042
	Yes	138	72	34.3	
Diabetes	No	212	79	27.1	0.035
	Yes	61	38	38.4	
Smoking history	Never	87	37	29.8	0.001
	Current	154	49	24.1	
	Ex-smoker	34	31	47.7	
Current smoker	No	121	68	36.0	0.011
	Yes	154	49	24.1	
Number of SMuRFs	0	27	5	15.6	0.079*
	1	96	38	28.4	
	2	87	41	32.0	
	3	48	28	36.8	
	4	14	5	26.3	
Any SMuRFs	No	27	5	15.6	0.067
	Yes	248	112	31.1	
Number of three SMuRFs (excluding current smoking)	0	70	17	19.5	0.002*
	1	111	43	27.9	
	2	65	39	37.5	
	3	26	18	40.9	
Any of the three SMuRFs (excluding current smoking)	No	71	17	19.3	0.014
	Yes	204	100	32.9	
Current smoking and any other three SMuRFs status	No SMuRFs	27	5	15.6	0.002
	Current smoker but no other SMuRFs	44	12	21.4	
	≥1 other SMuRFs but not current smoker	94	63	40.1	
	≥11 other SMuRFs and current smoker	110	37	25.2	
Smoking history and any other three SMuRFs status	Never smoked, no other SMuRFs	19	5	20.8	<0.001
	Never smoked, ≥1 other SMuRF	68	32	32.0	
	Current smoker, no other SMuRFs	44	12	21.4	
	Current smoker, ≥11 other SMuRFs	110	37	25.2	
	Ex-smoker, no other SMuRFs	8	0	0.0	
	Ex-smoker, ≥11 other SMuRFs	26	31	54.4	

* indicates that Mann Whitney test was used.

**Table 1B:** Patient characteristics by VT at EPS status (continuous variables).

Variable	No VT at EPS			VT at EPS			Mann–Whitney *p*-value
	Median	LQ	UQ	Median	LQ	UQ	
Age	56.0	49.0	65.0	58.0	52.0	66.0	0.245
LVEF (%)	34.0	30.0	37.0	31.0	26.0	36.0	0.002

The non-SMuRF variables demonstrating a univariable association with VT at EPS at the *p *≤ 0.2 level [namely, LVEF (%), gender, previous CABG status, and previous PTCA status] were selected as candidates for inclusion along with either the number of SMuRFs (0–4) or the presence of any SMuRFs in multiple logistic regression models. Table 2 shows the ORs for VT at EPS with 95% CIs for (a) each unit increase in the number of SMuRFs and (b) the presence of any SMuRFs adjusted for the independent predictors LVEF (%) and previous PTCA. The only independent predictors of VT at EPS were previous PTCA and LVEF (%).

**Table 2:** Multiple logistic regression models with the number of SMuRFs or any SMuRFs.

Model	Variable	Adjusted odds ratio	95% CI for adj OR		Adjusted *p*-value
			Lower	Upper	
(a)	Previous PTCA	1.8	1.0	3.2	0.050
	Per 10% decrease in LVEF (%)	1.8	1.3	2.5	0.001
	Per unit increase in number of SMuRFs (0–4)	1.1	0.9	1.4	0.226
(b)	Previous PTCA	2.0	1.1	3.7	0.031
	Per 10% decrease in LVEF (%)	1.8	1.3	2.5	0.001
	Any SMuRFs	2.5	0.9	6.8	0.074

When multiple logistic regression models were fitted incorporating LVEF (%), gender, previous CABG status, and previous PTCA status along with smoking history and either the number of other SMuRFs (0–3, excluding current smoking status) or the presence of any other SMuRFs as candidates for inclusion, the independent predictors of VT at EPS in the best-fitting model were LVEF (%), previous PTCA, smoking history, and any other SMuRFs (0–3 excluding current smoking status). The fit of this model was significantly better than that based on only LVEF (%) and previous PTCA [change in −2 × log (likelihood was 17.3, chi-squared with 3 degrees of freedom, *p* < 0.001)]. Table 3 shows the adjusted ORs with 95% CIs for VT at EPS in this best-fitting model.

**Table 3:** Multiple logistic regression models with smoking and other SMuRFs separated.

Variable	Adjusted odds ratio	95% CI for adj OR		Adjusted *p*-value
		Lower	Upper	
Previous PTCA	1.7	0.9	3.2	0.098
Per 10% decrease in LVEF (%)	1.9	1.4	2.7	<0.001
Smoking history				0.004
Never smoked	1	Reference category		
Current smoker	0.7	0.4	1.2	0.221
Ex-smoker	2.0	1.1	3.8	0.033
Any of other SMuRFs (excluding current smoking)	1.9	1.0	3.4	0.043

A total of 410 patients who underwent EPS were followed up for a median of 5.4 years; 37 patients died during the follow-up period. In total, 196 patients were followed up for at least 5 years and 66 patients for at least 10 years. Tables 4A,B show the patient characteristics during the EPS by mortality status.

**Table 4A:** Patient characteristics by mortality status (categorical variables).

Variable	Values taken	Alive		Dead		Log-rank *p*-value
		*n*	%	*n*	%	
Sex	Female	64	17.2	4	10.8	0.302
	Male	309	82.8	33	89.2	
Family history of CVD	No	180	50.6	21	65.6	0.091
	Yes	176	49.4	11	34.4	
Previous IHD	No	279	77.5	19	59.4	0.028
	Yes	81	22.5	13	40.6	
Previous CABG	No	352	98.1	29	90.6	0.027
	Yes	7	1.9	3	9.4	
Previous PTCA	No	314	87.2	26	78.8	0.184
	Yes	46	12.8	7	21.2	
Previous CVA	No	350	97.5	29	90.6	0.040
	Yes	9	2.5	3	9.4	
LVEF* *≤ 30%	>30%	251	67.3	13	36.1	<0.001
	≤30%	122	32.7	23	63.9	
Elevated BP	No	185	51.4	19	59.4	0.412
	Yes	175	48.6	13	40.6	
High cholesterol	No	165	46.0	16	50.0	0.495
	Yes	194	54.0	16	50.0	
Diabetes	No	269	75.1	22	68.8	0.468
	Yes	89	24.9	10	31.3	
Smoking history	Never	117	32.5	7	21.9	0.398
	Current	184	51.1	19	59.4	
	Ex	59	16.4	6	18.8	
Current smoker	No	176	48.9	13	40.6	0.236
	Yes	184	51.1	19	59.4	
Number of SMuRFs	0	31	8.7	1	3.1	0.450
	1	120	33.6	14	43.8	
	2	118	33.1	10	31.3	
	3	72	20.2	4	12.5	
	4	16	4.5	3	9.4	
Any SMuRFs	No	31	8.6	1	3.1	0.286
	Yes	329	91.4	31	96.9	

**Table 4B:** Patient characteristics by mortality status (continuous variables).

Variable	Alive		Dead		HR with 95% CI	*p*-value
	Median	(LQ, UQ)	Median	(LQ, UQ)		
Age	57	(50, 65)	60	(53, 71)	1.27 (0.95–1.69) per 10-year increase in age	0.106
LVEF (%)	33	(29, 37)	29	(24, 35)	1.91 (1.23–2.99) per 10% decrease in LVEF	0.004

The non-SMuRF variables demonstrating a univariable association with mortality at the *p *≤ 0.2 level [namely, age, LVEF (%), family history of CVD, previous IHD, previous CABG, previous PTCA, and previous CVA status] were selected as candidates for inclusion along with either the number of SMuRFs (0–4) or the presence of any SMuRFs in multiple Cox PH models of the right-censored outcome years from EPS to death. The only independent predictors were previous IHD, age, and LVEF (%). Table 5 shows the HRs with 95% CIs for (a) each unit increase in the number of SMuRFs and (b) the presence of any SMuRFs adjusted for the independent predictors age, LVEF (%), and previous IHD.

**Table 5:** Multiple Cox proportional hazards models for mortality.

Model	Variable	Hazards ratio	95% CI for HR		*p*-value
			Lower	Upper	
(a)	Per 10-year increase in age	1.3	1.0	1.8	0.077
	Per 10% decrease in LVEF (%)	1.9	1.2	3.1	0.009
	Previous IHD	2.0	1.0	4.1	0.064
	Per unit increase in the number of SMuRFs	1.0	0.7	1.4	0.888
(b)	Per 10-year increase in age	1.3	1.0	1.8	0.064
	Per 10% decrease in LVEF (%)	1.9	1.2	3.1	0.009
	Previous IHD	1.9	0.9	3.9	0.082
	Any SMuRFs	2.8	0.4	20.7	0.312

For every 10-year increase in age, the risk of death increased by a multiplicative factor of 1.3. For every 10% decrease in LVEF (%), the risk of death increased by a multiplicative factor of 1.9, and in patients with previous IHD, the risk of death was doubled. Neither the number of SMuRFs nor the presence of any SMuRFs was associated with mortality.

This study highlights the following significant results:

• Patients who are ex-smokers or have any other SMuRFs apart from smoking have double the odds of having inducible VT at EPS early after STEMI.
• Neither the presence of any SMuRFs nor the number of SMuRFs was associated with an increased risk of mortality.

## Discussion

In this study, we were able to examine potential differences in susceptibility to ventricular arrhythmias in individuals who presented with atherosclerotic MI in the absence of traditional risk factors. This subgroup of MI patients had been observed to have cardiac arrest at presentation and an excess mortality rate in the first 30 days (1–3), with most of this difference driven in the first 48 h and not explained by recurrent MI, heart failure, or stroke (1). However, our findings contradicted the hypothesis that STEMI patients without SMuRFs may be more sensitive to inducible VT, clearly demonstrating lower rates than those observed in patients with at least one standard risk factor. The rigorous approach we have validated over the last decade strengthened our study (6, 8, 15–17).

The apparent contradiction of our findings, despite the observed higher rates of cardiac arrest and mortality in SMuRF-less STEMI patients compared with those with at least one risk factor, may be attributed to the timing of our EPS. The underlying biological mechanism of VT in the first 48 h is known to be different from that driving VT at later timepoints. Indeed, VT in the first 48 h is not strongly predictive of VT/VF beyond this timepoint (18). Our team is dedicated to conducting early EP stimulation studies to detect the long-term risk of sudden cardiac death to guide preventative strategies, including automated implantable cardioverter defibrillator (AICD) insertion (11, 13, 16, 19). In this study, the earliest time post-STEMI was 4 days, with a median of 9 days. In contrast, as reported by Figtree et al. (1), most deaths in the SWEDEHEART cohort occurred in the first 48 h, with a difference in cardiac arrest identified at the time of presentation. If individuals survived to 30 days, mortality was less in SMuRF-less STEMI patients vs. those with at least one risk factor.

In addition to this potential explanation, our study population significantly differs from that of the Figtree et al. (1) paper, which excluded patients with pre-existing coronary artery disease (CAD), while 23% of our population had previous CAD. We also had a group of higher-risk patients because we only included those with LVEF ≤40% early after MI. Furthermore, only 8% of our patient population had no SMuRFs, compared with 15% of the SWEDEHEART cohort.

In our study cohort of patients undergoing EPS early following STEMI, the current smoking status SMuRF was associated with a decreased risk of VT at EPS. Since we have defined the current smoking SMuRF as current smokers vs. never and ex-smokers, these findings are unsurprising, given our patient population. Nearly 50% of all ex-smokers had inducible VT, while a similar proportion of never-smokers (30%) and current smokers (25%) had induced VT. Furthermore, 54% of ex-smokers with inducible VT had at least one other SMuRF, while only 32% of never-smokers and 25% of current smokers did. When smoking history was considered, ex-smokers were identified at an increased risk of VT at EPS, while those who never smoked or were current smokers had a comparable risk, as seen in our multiple regressions model. However, we also found that the presence of any SMuRFs apart from the current smoker status also conferred a similarly increased risk of inducible VT.

SMuRFs have been shown to worsen cardiovascular morbidity and mortality. It has been shown that patients with hypertension have an increased frequency of ventricular tachyarrhythmias (20, 21). Hypertension in itself is not arrhythmogenic but likely exerts its potential through its effect of pressure overload on the ventricle (22). Hypertension is a known risk factor for sudden cardiac death (SCD), especially with increasing LV mass (23). Diabetes can lead to myocardial dysfunction through mechanical abnormalities and electrical remodeling (24). Arrhythmogenesis is also exacerbated in patients with diabetes because of autonomic dysregulation (25) and inflammation (26). The AVID study (27) has previously shown that lipid-lowering therapy had a positive impact on decreasing ventricular arrhythmia in patients with atherosclerotic heart disease. Smoking has been shown to have deleterious effects on the myocardium with the predisposition to an arrhythmogenic substrate in an animal model (28). Smoking cessation improves cardiovascular outcomes, but it can take up to 5 (29) and even 15 (30) years for outcomes to stabilize to levels of never-smokers. The effect of previous smoking may confound the data as we did not have data on the time of smoking cessation and the number of cigarettes consumed.

We have previously confirmed the utility of the inducibility of VT in predicting future arrhythmic events (6, 7, 10). Conversely, patients with a negative EPS, meaning no induced arrhythmia or ventricular flutter/VF, have demonstrated a good long-term prognosis without an ICD insertion (8, 13). Confirming an EPS-guided strategy for the primary prevention of sudden cardiac death requires a large, multicenter, randomized, controlled trial such as the current PROTECT-ICD trial, which has a VT induction protocol similar to our study (31). Gatzoulis et al. have also shown the utility of EPS after STEMI in a population with LVEF >40% (32).

The limitations of this study include the lack of randomization and the small sample size from a single center. Although we have used the same definitions from the seminal Lancet paper (1) to define SMuRFs, we acknowledge that individual-level characterization of each risk factor would enhance the results. Characterizing the infarct substrate using late gadolinium enhancement on cardiac magnetic resonance imaging may provide additional and novel insights into this high-risk population. Although a relatively small study sample was analyzed, statistically significant results regarding VT inducibility were obtained. The routine use of EPS shortly after MI to guide ICD implantation is part of a study protocol and is limited by its invasiveness and costs. However, it might offer a rational and cost-effective approach to the expensive long-term therapy with ICD following MI. This approach of using EPS early after MI in individuals with impaired left ventricular function is under investigation in the randomized PROTECT ICD trial (31).

## Conclusions

In our cohort of patients with LVEF ≤40% early after STEMI who had an EPS to assess for inducible VT at a median of 9 days, either the presence of any of the three SMuRFs (hypertension, hypercholesterolemia, or diabetes mellitus) or previous smoking doubled the chance of having inducible VT at EPS. Neither the presence of any SMuRFs nor the number of SMuRFs was associated with an increased mortality risk on long-term follow-up. These results further highlight the need to modify SMuRFs in a high-risk subset of patients with IHD to reduce the significant risk of ventricular tachyarrhythmias following MI.

## Data Availability

The raw data supporting the conclusions of this article will be made available by the authors, without undue reservation.
