# Supplementary material for: Influence of standard modifiable risk factors on ventricular tachycardia after myocardial infarction
Source: Front Cardiovasc Med. 2023 Oct 24;10:1283382. doi: 10.3389/fcvm.2023.1283382 (PMC10628449; doi:10.3389/fcvm.2023.1283382)
Supplement: Supplementary file 1 [file Datasheet1.pdf]

Programmed ventricular stimulation

Programmed ventricular stimulation was performed at twice diastolic threshold at the right ventricular apex (single site) using a programmable stimulator. Within programmed ventricular stimulation, a drive train (S1) of 8 beats at 400ms was followed by up to 4 extrastimuli. Each extra-stimulus was introduced at 300ms coupling interval and decremented at 10ms interval until refractory. Once an extra-stimulus was refractory, its coupling interval was increased by 10ms so that it was able to capture the ventricle again and the next extra-stimulus was introduced at 300ms and the process of decrementing the coupling interval repeated until that extra-stimulus was also refractory. There was no set minimum cut-off for each extra-stimulus. This process was continued until the fourth extra-stimulus was refractory or ventricular tachyarrhythmia was induced.

If the first programmed ventricular stimulation was positive for inducible ventricular tachycardia, the study was stopped and further programmed ventricular stimulation inductions were not performed. However, as ventricular tachycardia can be induced on second programmed ventricular stimulation when negative on the first in a significant proportion of post-myocardial infarction patients with left ventricular dysfunction, the same electrophysiology study protocol was repeated after a period of 5–10 minutes if ventricular tachycardia was not induced during the first programmed ventricular stimulation at the same site. Isoproterenol infusion was not utilized for programmed ventricular stimulation.

Definition of SMuRFs

A patient was defined as a current smoker if they had regularly smoked (≥1 cigarette per day) within the past month before the index hospitalisation. Hypercholesterolaemia was defined as having a previous diagnosis of hypercholesterolaemia, previous or ongoing oral LDL

cholesterol (LDL-C) lowering treatment, an LDL-C concentration of 3.5 mmol/L or higher, or a total cholesterol concentration of 5.5 mmol/L or higher during the index admission. Diabetes (type 1 and type 2) was defined as having a previous diagnosis of diabetes or previous glucose lowering pharmacotherapy. Hypertension was defined as having a previous diagnosis of hypertension or previous antihypertensive pharmacotherapy, or a new diagnosis of hypertension during the index admission.
